# Handedness did not affect motor skill acquisition by the dominant hand or interlimb transfer to the non-dominant hand regardless of task complexity level

**DOI:** 10.1038/s41598-022-21962-2

**Published:** 2022-10-28

**Authors:** János Négyesi, Péter Négyesi, Tibor Hortobágyi, Sai Sun, Joji Kusuyama, Rita M. Kiss, Ryoichi Nagatomi

**Affiliations:** 1 Division of Biomedical Engineering for Health and Welfare, Tohoku University Graduate School of Biomedical Engineering, Tohoku University, 2-1, Seiryo-machi, Aoba-ku, Sendai, 980-8575 Japan; 2 Doctoral School of Education, Eszterházy Károly Catholic University, Eger, Hungary; 3 Department of Kinesiology, Hungarian University of Sports Science, Budapest, Hungary; 4 Institute of Sport Sciences and Physical Education, University of Pécs, Pécs, Hungary; 5 Somogy County Kaposi Mór Teaching Hospital, Kaposvár, Hungary; 6 Center for Human Movement Sciences, University of Groningen, University Medical Center Groningen, Groningen, The Netherlands; 7 Frontier Research Institute for Interdisciplinary Sciences, Tohoku University, Sendai, Japan; 8 Research Institute of Electrical Communication, Tohoku University, Sendai, Japan; 9 Department of Mechatronics, Optics and Engineering Informatics, Faculty of Mechanical Engineering, Budapest University of Technology and Economics, Budapest, Hungary; 10 Department of Medicine and Science in Sports and Exercise, Tohoku University Graduate School of Medicine, Sendai, Japan

**Keywords:** Cognitive neuroscience, Learning and memory, Motor control

## Abstract

Patients undergoing unilateral orthopedic or neurological rehabilitation have different levels of impairments in the right- or left-dominant hand. However, how handedness and the complexity of the motor task affect motor skill acquisition and its interlimb transfer remains unknown. In the present study, participants performed finger key presses on a numeric keypad at 4 levels of sequence complexities with each hand in a randomized order. Furthermore, they also performed motor sequence practice with the dominant hand to determine its effect on accuracy, reaction time, and movement time. The NASA-TLX at the end of each block of both testing and practice was used to confirm participants’ mental workload related to sequence complexity. Both right- and left-handed participants performed the motor sequence task with faster RT when using their right hand. Although participants had increasing RT with increasing sequence complexity, this association was unrelated to handedness. Motor sequence practice produced motor skill acquisition and interlimb transfer indicated by a decreased RT, however, these changes were independent of handedness. Higher sequence complexity was still associated with longer RT after the practice, moreover, both right- and left-handed participants’ RT increased with the same magnitude with the increase in sequence complexity. Similar behavioral pattern was observed in MT as in RT. Overall, our RT results may indicate left-hemisphere specialization for motor sequencing tasks, however, neuroimaging studies are needed to support these findings. On the other hand, handedness did not affect motor skill acquisition by the dominant hand or interlimb transfer to the non-dominant hand regardless of task complexity level.

## Introduction

Motor learning aims to facilitate the acquisition of novel motor skills or help relearn skills impaired or abolished by an injury^1^. Although both hemispheres are involved in motor control, findings from visually guided motor task (visuomotor) studies suggest a functional interhemispheric asymmetry. While the left hemisphere is most dominantly involved in the temporal evaluation of visual stimuli^2,3^, the right hemisphere is dedicated to transforming the visual information to guide movements based on spatial recognition^4–6^. Consequently, visuospatial processing is suggested to be driven by the right hemisphere^7^, while motor sequence learning is mainly driven by left hemisphere. Nevertheless, results from studies aimed to determine the interhemispheric differences in visuospatial processing during visuomotor tasks are controversial^8–10^.

Functions such as language, speech, or face recognition show localization to the right or left side of the brain. This phenomenon is called hemispheric lateralization. Ninety percent of healthy adults are right-hand dominant and, therefore, perform fundamental manual motor tasks with the right hand^11–13^. The nature of side-dominance is a consequence of (1) the evolutionary specialization of the left hemisphere for skilled motor activities^14–16^, and (2) brain lateralization through complex motor control processes (for reviews, see^17,18^). While it was suggested for decades that left-handers have lower brain lateralization than right-handers, i.e., some cognitive and motor functions of left-handers are distributed more evenly across the left and right cerebral hemispheres, recent motor behaviour studies show equivalent asymmetries between left- and right-handers (for review, see^19^).

Handedness also seems to affect motor skill acquisition: while right-handed people had significantly greater skill improvements for their dominant-right hand compared to their non-dominant left hand, left-handers demonstrated comparable learning effects in each hand^20^. Wang et al.^21^ detected differences in the magnitude of motor skill acquisition between left- and right-handed participants of not only the trained limb but also in the untrained contralateral limb. This phenomenon is called interlimb transfer^22^ (also known as cross-education) and is known to be regulated at multiple levels of the nervous system including cortical, subcortical, and spinal networks^23,24^. Clinical studies reported a significant decline in motor skills after left but not right hemispheric damage^25^ supporting the idea of a left-hemisphere dominance for motor sequencing tasks. Moreover, while some studies reported that the magnitude of interlimb transfer might be affected by the degree of handedness^26–28^, others failed to detect such an effect^29,30^.

Hemispheric asymmetry is also associated with task complexity, i.e., the left hemisphere has been associated with higher-order aspects of motor control, supported by clinical studies^31–33^. Moreover, an fMRI study^34^ suggests that ipsilateral motor cortex activity in unimanual tasks differs between left-handed and right- handed individuals. This activity is related to task complexity according to the execution of the task with movements involving one finger, multiple fingers, or sequential finger movements. During the practice of a motor sequence, participants coordinate the order of finger movements. A previous study^35^ reported different patterns of hemispheric lateralization in response to motor sequence practice with different complexity. Although this study also reported different activation patterns after motor learning with the dominant- versus non-dominant hand, none of the previous studies have examined the interaction between handedness and the complexity of the motor sequence task.

Therefore, the primary objective of the study is to reveal a potential interaction between handedness and the complexity of the motor sequence task. Based on the preponderance of literature suggesting motor functions of left- versus right-handers are distributed more evenly across the two hemispheres, we hypothesized that a dominant-hand practice would result in a higher magnitude of motor skill acquisition in right-handed participants. While the role of dominant versus non-dominant hand in cross-education has been examined^36–39^, the role of right versus left-hand dominance in cross-education is unclear, therefore, proposing a directional hypothesis at this stage is premature. However, we hypothesize that the nature of the differences between the two hands might be related to the level of task complexity considering that task complexity increases cognitive demand. To address this hypothesis, participants in this study performed finger key presses on a numeric keyboard with each hand in separate trials at 4 levels of complexity. Moreover, participants underwent a short-term dominant-hand motor sequence practice, and then the changes in the dependent variables were measured in both the trained (motor skill acquisition) and non-trained (interlimb transfer) hands. To evaluate the interaction between handedness and motor sequencing complexity, we analyzed the accuracy, reaction time, and movement time of each trial. Notably, the National Aeronautics and Space Administration Task Load Index (NASA-TLX) to determine if participants’ subjective rating of task complexity aligned with the experimentally designed sequence complexity.

## Materials and methods

### Participants

The sample size was based on an a priori power analysis performed in G*Power (version 3.1.9.3^40^) assuming a type I error of 0.05 and statistical power of 0.80. We determined effect sizes based on behavioural data of a previous study^21^, which aimed to determine the differences in the magnitude of motor skill acquisition and interlimb transfer between left- and right-handed participants after short-term unilateral motor skill practice. To ensure that the sample size in the present study would be sufficient to detect significant differences between the 2 groups (left-handed, right-handed) and the 2 measurements (pre, post), we chose the lowest partial eta squared value from the previous study (time × hand × group interaction, η_p_^2^ = 0.32) as input for the power analysis.

Considering potential drop-outs, we recruited 12 strongly left- (mean ± SD, age = 30.5 ± 6 years, height = 172.8 ± 6.2 cm, body mass = 67.7 ± 10.7 kg) and 13 strongly right-handed (age = 26.5 ± 7.7 years, height = 174.3 ± 9.6 cm, body mass = 68.1 ± 14.7 kg) participants with no reported neurological deficits or sensorimotor impairment. Handedness was determined using the Edinburgh Handedness Inventory^41^, a scale that is used to measure the degree of hand laterality in daily activities such as writing, drawing, throwing, using scissors, brushing teeth, opening a box, striking a match and using a pair of scissors knife, spoon, and a broom. Laterality index was calculated by summing the number of tasks performed with the right limb and the number of tasks performed with the left limb (L) as follows: (R − L)/(R + L). Laterality index for left- and right-hand dominant participants was − 89.1 ± 11.4 and 88.4 ± 11.5, respectively. After giving both verbal and written explanations of the experimental protocol, participants signed the informed consent document in accordance with the declaration of Helsinki. All experimental protocols were approved by the University (Hungarian University of Sports Science, Budapest, Hungary) Ethical Committee (Approval No. TE-KEB/No1/2021).

### Experimental procedures

**Figure 1:**
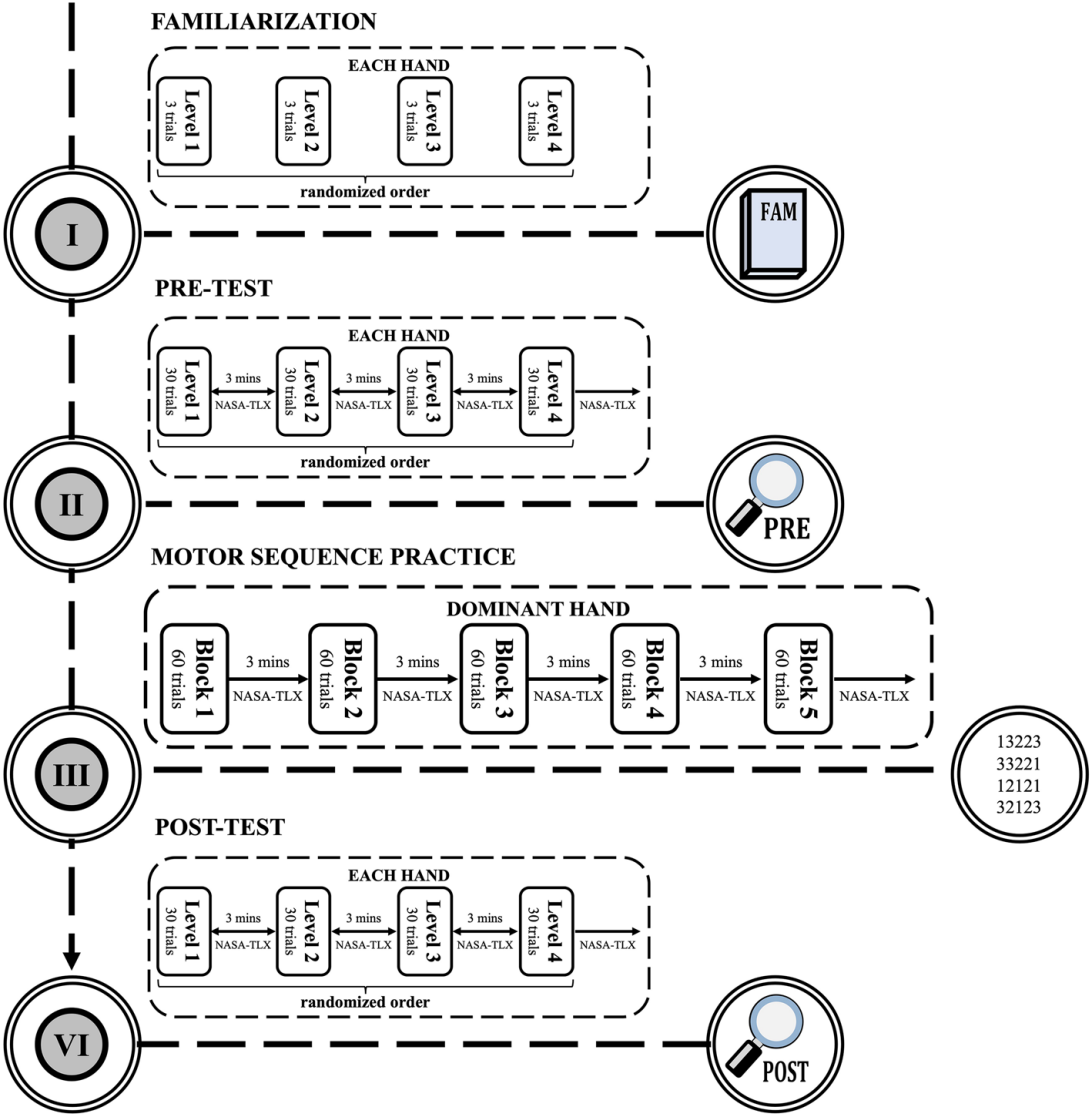
Schematic illustration of the experimental procedures. Participants performed a familiarization of each sequence complexity with each hand before the start of testing. Participants were instructed to perform finger key presses in response to numeric sequences presented on the screen with both their right and left hand in a randomized order. The test consisted of 4 blocks of 30 trials with different sequence complexity. After the pre-test, participants performed the motor sequence practice with their dominant hand. The practice was divided into 5 blocks of 60 trials. Immediately after the practice, participants performed the post-test, which consisted of the same set of sequences in each block as the pre-test, however, the order of appearance was randomized. The NASA-TLX was administered at the end of each block of both testing and practice. Images within the figure were drawn by János Négyesi using Microsoft Office Power Point 2018 software (Microsoft, Redmond, Washington, USA).

Figure 1 depicts the experimental procedures. After the familiarization trials, first, participants performed finger key presses with each hand in a randomized order in response to numeric sequences presented on the monitor placed in front of them. The pre-test consisted of 4 blocks of 30 trials with different sequence complexity in a randomized order. After each block, NASA-TLX was administered to determine if participants’ subjective rating of task complexity aligned with the experimentally designed sequence complexity. Next, participants performed the motor sequence practice consisting of 5 blocks of 60 trials with their dominant hand. After each block, participants performed the NASA-TLX. After practice, participants received the same test sequence they performed before practice. Participants received 5 min of rest between the test and practice trials.

### Motor sequence tests

**Figure 2:**
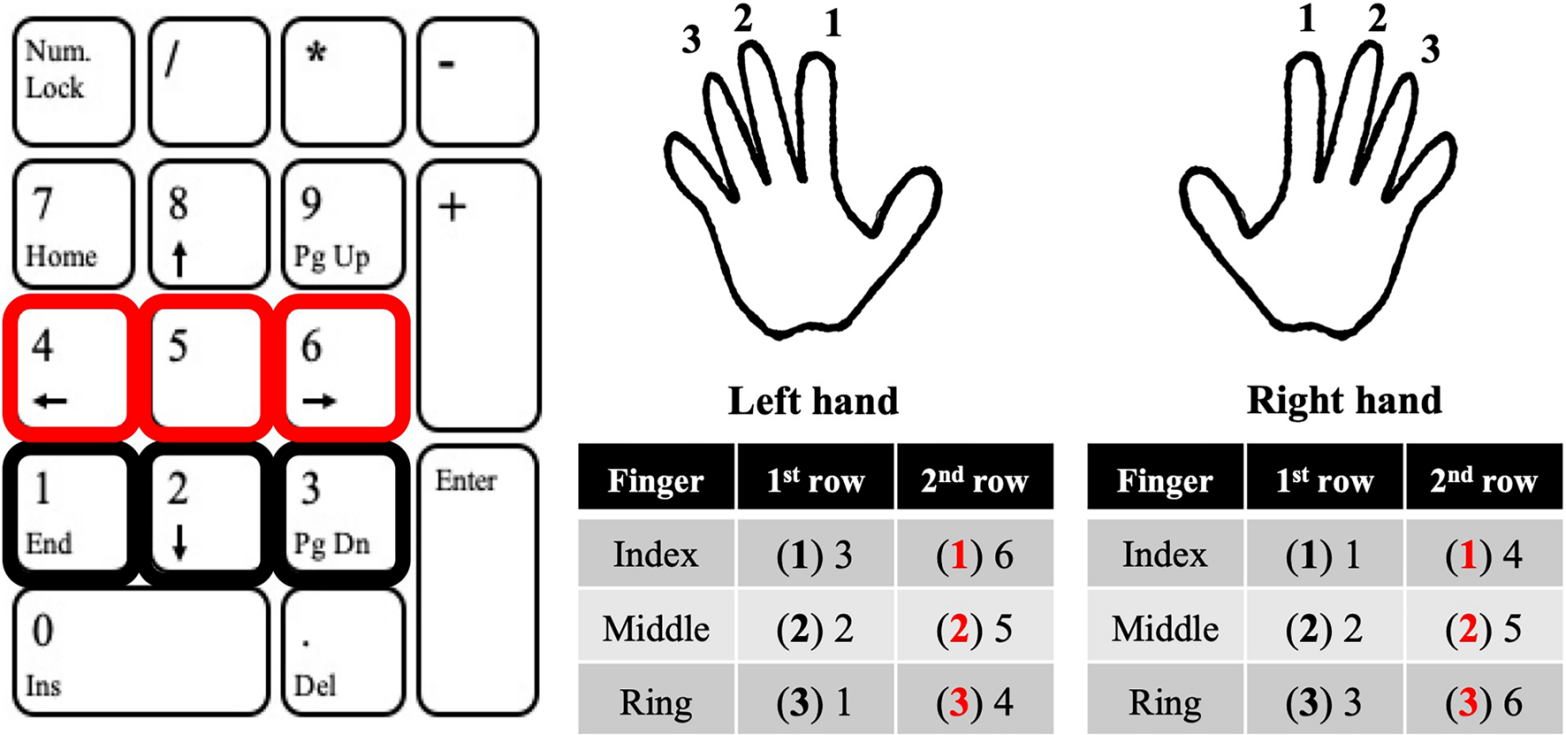
Experimental setup of the numeric keypad. The index (1), middle (2), and ring (3) fingers of the right or left hand were placed on the numeric keyboard’s lower line, covered with a virtual partition. The digits 1, 2, and 3 corresponded to ‘1’, ‘2’, and ‘3’ keys for the right hand and to ‘3’, ‘2’, and ‘1’ keys for the left hand. For the 3rd and 4th conditions, the second line of the numeric keyboard was also involved and was indicated with red numbers on the screen. In this case, the digits 1, 2, and 3 corresponded to ‘4’, ‘5’, and ‘6’ keys for the right hand and to ‘6’, ‘5’, ‘4’ keys for the left hand. Images within the figure were drawn by János Négyesi and were scanned using Microsoft Office Lens application (Microsoft, Redmond, Washington, USA).

Participants performed finger key presses with each hand in a randomized order in response to numeric sequences presented in the middle of an LCD monitor with a display resolution of 1.024 × 768 pixels and a refresh rate of 60 Hz. The size of the numeric sequences was nearly a quarter of the screen. The index (1), middle (2), and ring (3) fingers of the right or left hand were placed on the numeric keyboard’s lower line, covered with a virtual partition. The digits 1, 2, and 3 corresponded to ‘1’, ‘2’, and ‘3’ keys for the right hand and to ‘3’, ‘2’, and ‘1’ keys for the left hand. For the 3rd and 4th conditions, the second line of the numeric keyboard was also involved and was indicated with red numbers on the screen. In this case, the digits 1, 2, and 3 corresponded to ‘4’, ‘5’, and ‘6’ keys for the right hand and to ‘6’, ‘5’, ‘4’ keys for the left hand (Fig. 2). A trial consisted of the performance of one sequence, including five movements (digits). Supplementary Fig. 1 shows the motor sequence trials for the 4 blocks with different sequence complexity. Each block consisted of 30 trials in a randomized order. Prior to each trial, participants looked at the center of the blank white screen. Participants were instructed to immediately perform the sequence as quickly and accurately as possible after the appearance of a five-digit number sequence presented on the screen. Familiarization with the motor sequence tasks of each sequence complexity consisted of three trials with each hand before the start of testing. Each participant received the same set of sequences in each block before and after the motor sequence practice, however, the order of appearance was randomized. At the end of each block, the NASA-TLX was administered (see below). Completing the questionnaire required ~ 3 min.

### Motor sequence practice

Motor sequence practice consisted of 300 trials performed with the participants’ dominant hand. Each trial involved only one finger but might require the transition between the first and second row of the numeric keyboard. These trials were different from the test trials and were divided into 5 blocks of 60 trials. Supplementary Fig. 2 summarizes the trials for each block. The NASA-TLX was administered at the end of each block. Moreover, after every 15 trials, participants were asked to count backward by seven, starting from a randomly determined two-digit number to keep their attention high.

### National aeronautics and space administration task load index (NASA-TLX)

The NASA-TLX was administered at the end of each block of the motor sequence tests (4–4 blocks for pre and post-test) and practice (5 blocks) to determine if participants’ subjective rating of task complexity aligned with the experimentally designed sequence complexity^42,43^. Participants rated six dimensions (Supplementary Fig. 3) related to perceived effort completing the motor task with their assigned complexity on a scale from 0 to 100: mental demand, physical demand, temporal demand, own performance, effort, and frustration. To determine the weighting of each dimension, participants completed pairwise comparisons across all pairs of the six dimensions (Supplementary Fig. 4). Weightings were given to each dimension based on the number of times a dimension was chosen as most relevant. Total workload scores were computed by multiplying the weighting with the rating score of each dimension, summing the scores across all dimensions, and dividing by 15^44^.

### Data analyses

The dependent variables were accuracy (percent of correct trials), reaction time (RT), and movement time (MT) and were determined using the custom-made software. We measured RT to characterize system integration between sensory input and motor output and we used MT to characterize the execution element of the task. A trial was defined as correct if all five key presses were executed in the specified order. RT is defined as the difference in time between the stimulus onset on the screen and the first keypress, while MT is the time between the end of the RT interval and the last keypress. We analyzed only the correct trials’ RT and MT, trials with pressing the wrong key, or pressing two or more keys simultaneously were automatically excluded from the data and statistical analyses.

### Statistical analyses

Statistical analyses were performed using SPSS Statistics Package (version 22.0, SPSS Inc., Chicago, IL). Data were checked for normal distribution using the Shapiro–Wilk test and homogeneity of variances using Levene’s test. To statistically investigate the differences between left and right-handed participants’ accuracy, RT, and MT of the motor sequence test, a series of handedness (left-handed, right-handed) × sequence complexity (levels 1–4) × hand (dominant, non-dominant) mixed analysis of variance (ANOVA) and planned post-hoc tests with Bonferroni correction for multiple comparisons were performed. To detect the effect of a dominant-hand motor sequence practice on this interaction, separate handedness × time (pre, post) × sequence complexity mixed ANOVAs with post-hoc tests were conducted for both motor skill acquisition (dominant hand) and interlimb transfer (non-dominant hand). Moreover, separate mixed ANOVA was performed to assess differences between LH and RH participants’ NASA-TLX scores at each time-point, hand, and sequence complexity level. Lastly, separate mixed ANOVAs were also performed to determine the changes in the dependent variables, including the NASA-TLX data, throughout the 5 blocks of the motor practice. Compound symmetry was evaluated with the Mauchly's test and the Greenhouse–Geisser correction and was used when required. When data violated the assumption of sphericity so that when the Epsilon was less than 0.75 for Mauchly’s test of sphericity, we used the Greenhouse–Geisser-corrected value and the Huynh–Feldt-corrected value for epsilon greater than 0.75. Complementary post-hoc analyses (repeated measures ANOVAs and paired-samples t-tests) were used when indicated. Cohen’s effect size, d, was also computed as appropriate. Additionally, the effect sizes of the independent variables were expressed using partial eta squared (η_p_^2^)^45^. In order to characterize the relationship between behavioral metrics and subjective workload, a series of Pearson’s correlation analyses were performed. Statistical significance was set at *p* < 0.05.

### Ethics statement

After giving both verbal and written explanations of the experimental protocol, participants signed the informed consent document in accordance with the declaration of Helsinki. All experimental protocols were approved by the University (Hungarian University of Sports Science, Budapest, Hungary) Ethical Committee (Approval No. TE-KEB/No1/2021).

## Results

Each analyses of MT revealed similar gross patterns of changes as RT, moreover, the two behavioral measure correlated with each other at each sequence complexity level. Therefore, only the results of accuracy and RT are reported and presented in Figures. Results of statistical analyses in MT can be found in Supplementary Table 1 and Supplementary Fig. 5. Notably, we report the correct trials’ RT and MT, however, similar findings were found when all trials (both correct and incorrect) were included.

### Differences between left and right-handed participants’ motor sequence performance (pre-test results)

Mixed ANOVA showed a main effect of sequence complexity (F_3,21_ = 18.457, *p* < 0.001, η_p_^2^ = 0.725) in NASA-TLX. Post-hoc analysis revealed that participants’ mental workload increased from level 1 to 2 (*p* = 0.001, d = 0.375) and level 3 to 4 (*p* < 0.001, d = 0.803), moreover, each sequence complexity level resulted in a higher mental workload as compared to level 1 (all *p* ≤ 0.004), regardless of handedness.

**Figure 3:**
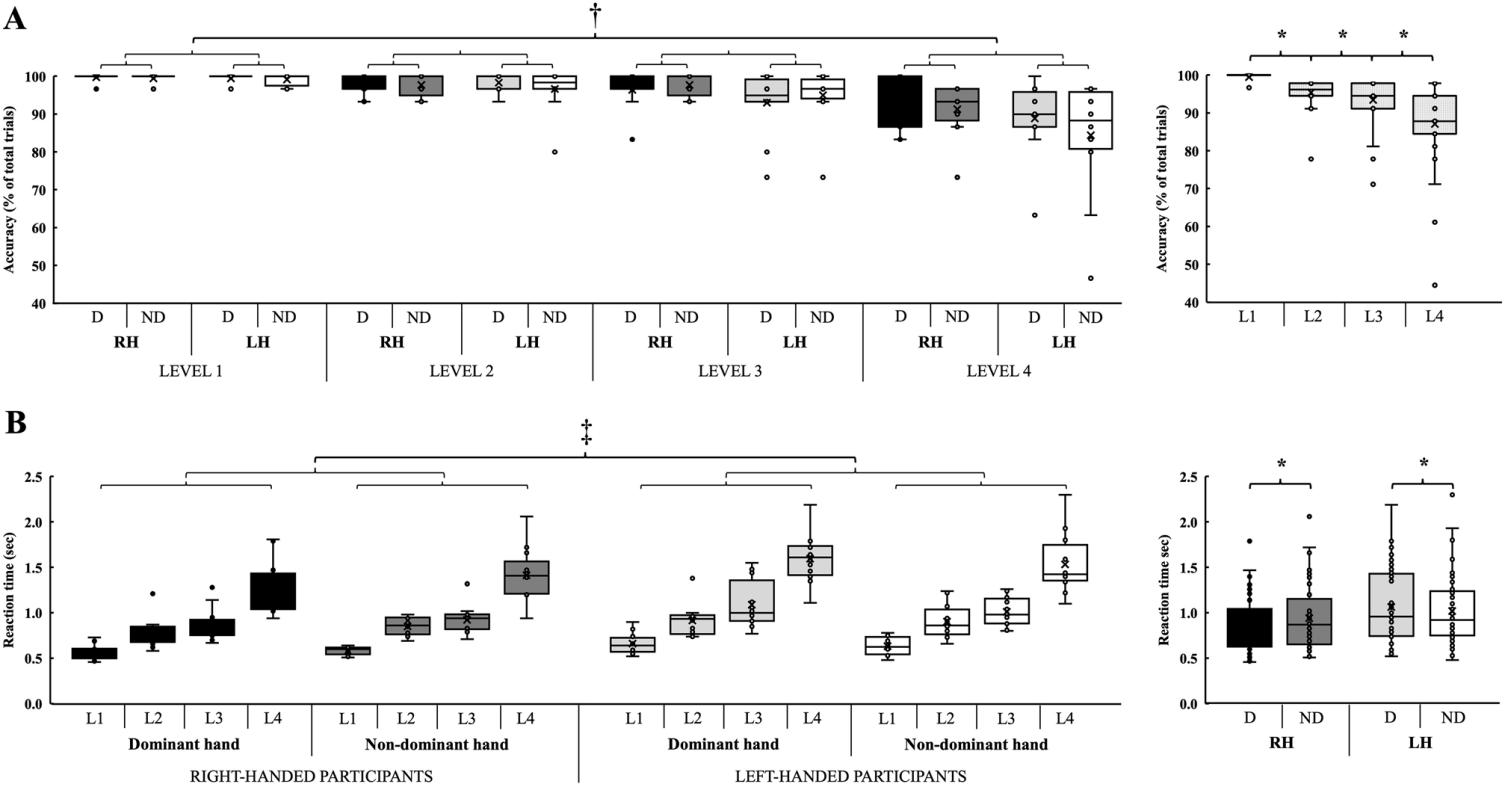
The effects of handedness and sequence complexity on accuracy and reaction time. Panel (**A**) Accuracy, determined by the percent of correct motor sequences decreased with the increase in sequence complexity, regardless of handedness. Panel (**B**) Both right- and left-handed participants performed the motor sequence task with less RT when using their right hand. D: dominant hand; L: sequence complexity level; LH: left-handed participants; ND: non-dominant hand; RH: right-handed participants The boxplots show the median, the upper, and lower quartiles, and the min and max value of the groups. “ × ” within the boxplot represents the mean line. ‡ handedness × hand interaction effect; † main effect of sequence complexity, * *p* < 0.05.

Statistical analysis revealed a main effect of sequence complexity (F_3,21_ = 10.028, *p* < 0.001, η_p_^2^ = 0.589) in accuracy. Post-hoc analysis showed a decreasing percent of correct motor sequences with the increase in sequence complexity (1 > 2 > 3 > 4, all *p* ≤ 0.05), regardless of handedness  (Fig. 3A). In addition, more errors at level 3 were associated with a higher mental workload (r = − 0.316, *p* = 0.026).

There was a main effect of sequence complexity (F_3,21_ = 341.489, *p* < 0.001, η_p_^2^ = 0.981) in RT with the post-hoc analysis showing increasing RT with increasing sequence complexity (each *p* ≤ 0.001), regardless of handedness. In addition, there was a handedness × hand interaction effect (F_1,23_ = 9.378, *p* = 0.006, η_p_^2^ = 0.290). Post-hoc analyses revealed that both right- and left-handed participants performed the motor sequence task with faster RT when using their right hand (*p* < 0.001, d = 0.192; *p* = 0.032, d = 0.114; respectively) (Fig. 3B). We found positive moderate correlations (0.381 ≤ r ≤ 0.562, each *p* ≤ 0.005) between mental workload and RT at each level, indicating that participants subjectively experienced a lower level of mental workload with the decrease in RT.

### Motor skill acquisition and interlimb transfer at different sequence complexity after dominant hand motor sequence practice (pre vs. post-test results)

Only a main effect of sequence complexity for both motor skill acquisition (F_3,21_ = 27.195, *p* < 0.001, η_p_^2^ = 0.795) and interlimb transfer (F_3,21_ = 29.010, *p* < 0.001, η_p_^2^ = 0.806) was found in NASA-TLX. Post-hoc tests revealed an increasing mental workload with the increase in sequence complexity between each level for the practicing dominant hand (1 < 2 < 3 < 4, each *p* ≤ 0.002) and between levels 2 and 4 (2 < 3 < 4, each *p* ≤ 0.001) for the non-practicing non-dominant hand, regardless of handedness and time.

**Figure 4:**
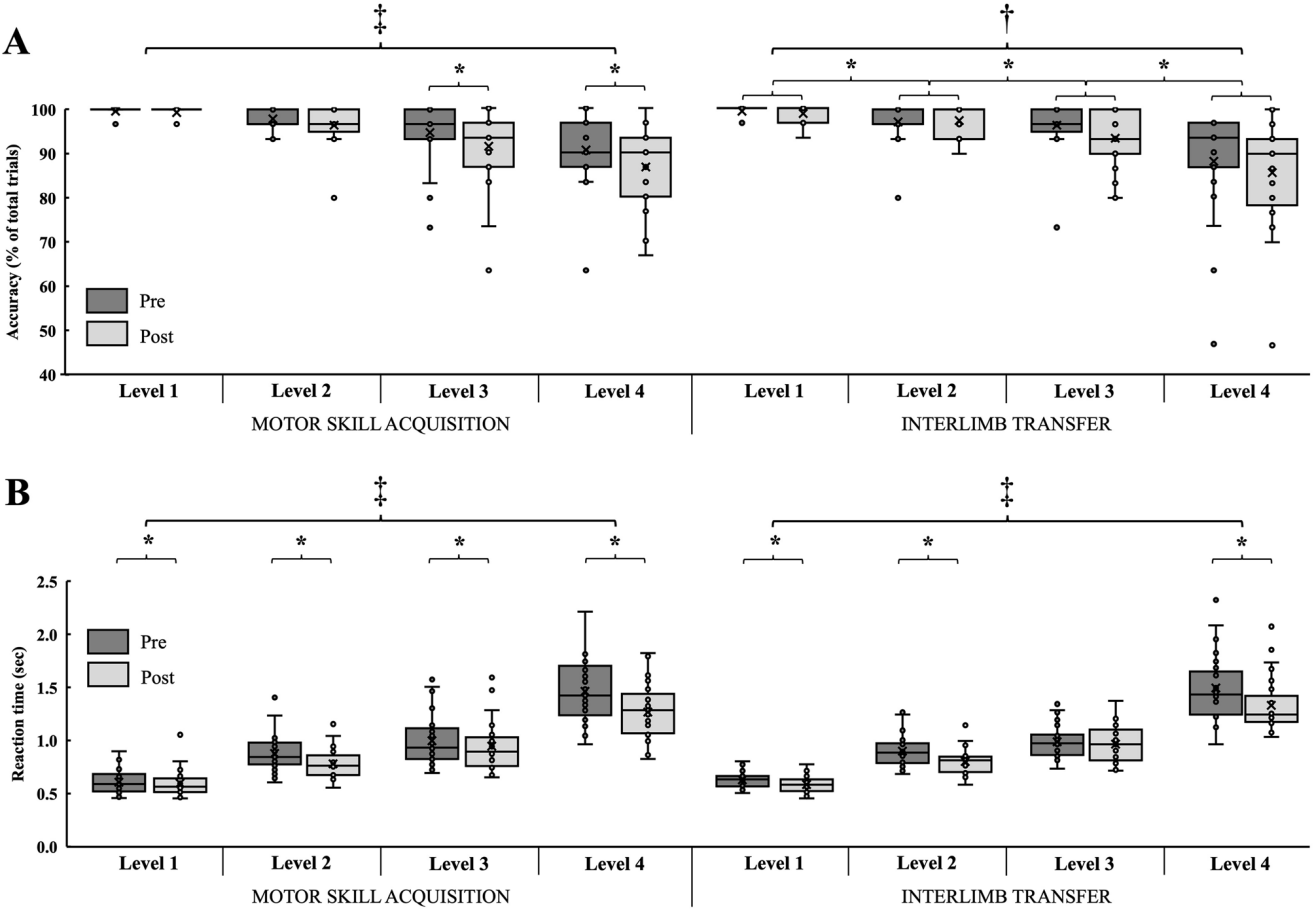
Motor skill acquisition and interlimb transfer at different sequence complexities after dominant hand motor sequence practice. Panel (**A**) Participants performed the motor sequence tasks with less accuracy at level 3 and level 4 after the practice with their practicing dominant hand, regardless of handedness. Moreover, motor sequence errors increased with the increase in sequence complexity in the non-practicing hand. Panel (**B**) The practice produced motor skill acquisition indicated by a decreased RT at each level post versus pre-test. In addition, the motor skill practice also induced interlimb transfer of decreased RT at levels 1, level 2, and level 4, regardless of handedness. Results also suggest an interlimb transfer of decreased MT at each level, regardless of handedness. The boxplots show the median, the upper, and lower quartiles, and the min and max value of the groups. “ × ” within the boxplot represents the mean line. ‡ time × sequence complexity interaction effect; † main effect of sequence complexity, * *p* < 0.05.

The motor sequence practice induced changes in accuracy indicated by time (F_1,23_ = 17.796, *p* < 0.001, η_p_^2^ = 0.436) and sequence complexity (F_3,21_ = 14.852, *p* < 0.001, η_p_^2^ = 0.680) main effects and also a time × sequence complexity interaction effect (F_3,21_ = 4.875, *p* = 0.010, η_p_^2^ = 0.411). Post-hoc analyses revealed that participants performed the motor sequence tasks with less accuracy at level 3 (91.3 ± 8.5%, *p* = 0.006, d = 0.436) and level 4 (86.7 ± 8.4%, *p* = 0.017, d = 0.450) after the practice as compared to the pre-test (94.8 ± 6.6%, 90.5 ± 8%, respectively) (Fig. 4A). Nevertheless, accuracy and mental workload were significantly associated only at level 3 after the practice (r = − 0.509, *p* = 0.009). In addition, a main effect of sequence complexity (F_3,21_ = 9.987, *p* < 0.001, η_p_^2^ = 0.588) was found for the non-practicing hand with the post-hoc analysis showing increasing errors with the increase in sequence complexity (each *p* ≤ 0.019), regardless of time and handedness (Fig. 4A).

There were time (F_1,23_ = 30.946, *p* < 0.001, η_p_^2^ = 0.574) and sequence complexity (F_3,21_ = 256.431, *p* < 0.001, η_p_^2^ = 0.973) main effects and also a time × sequence complexity interaction effect (F_3,21_ = 5.934, *p* = 0.004, η_p_^2^ = 0.459) in RT of the dominant hand. Post-hoc analyses suggested that the practice produced motor skill acquisition indicated by a decreased RT at each level post versus pre-test (each *p* ≤ 0.019)  (Fig. 4B). The motor skill practice induced not only motor skill acquisition but also interlimb transfer of RT indicated by time (F_1,23_ = 44.194, *p* < 0.001, η_p_^2^ = 0.658) and sequence complexity (F_3,21_ = 247.452, *p* < 0.001, η_p_^2^ = 0.972) main effects and also a time × sequence complexity interaction effect (F_3,21_ = 6.803, *p* = 0.002, η_p_^2^ = 0.493). Post-hoc analyses showed that the practice induced a decreased RT in the non-dominant hand at level 1 (*p* < 0.001, d = 0.563), level 2 (*p* < 0.001, d = 0.746), and level 4 (*p* < 0.001, d = 0.580), regardless of handedness (Fig. 4B). However, changes in RT were not associated with participants’ subjective mental workload neither for the practicing dominant nor for the non-practicing non-dominant hand (all *p* > 0.05).

**Figure 5:**
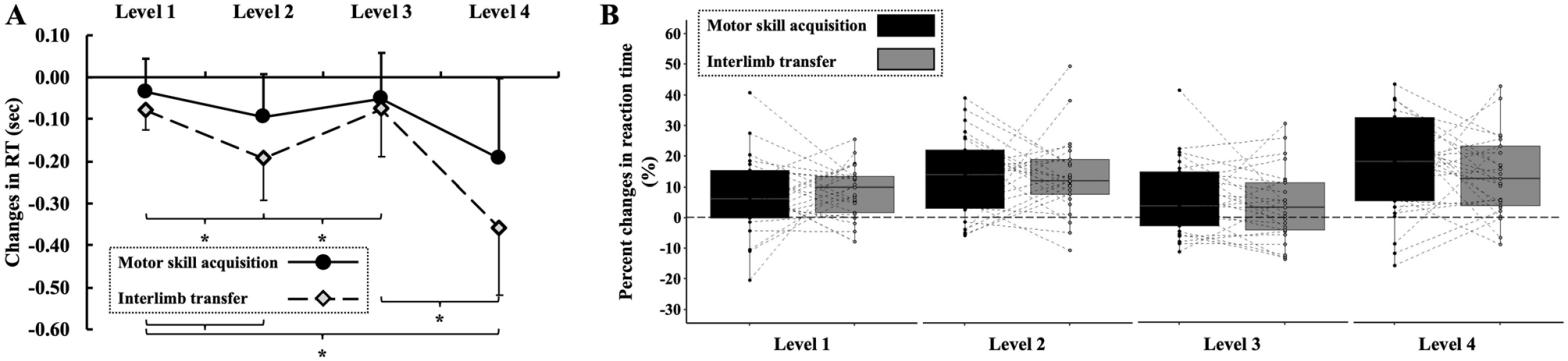
Changes in participants’ reaction time. Panel (**A**) Participants’ RT improved the most at the most complex finger tapping sequence task for both motor skill acquisition (black circles) and interlimb transfer (grey diamonds), regardless of handedness. Symbols and vertical bars denote mean values ± 1SD. Panel (**B**) No differences were found in the level of motor skill acquisition (black boxes) and interlimb transfer (grey boxes) at any complexity levels. Dotted lines between boxes represent the percent changes of motor skill acquisition and interlimb transfer of the same participant. Larger values than zero correspond to better post-intervention reaction times as compared to baseline values. The boxplots show the median, the upper, and lower quartiles, and the min and max value of the groups. “ × ” within the boxplot represents the mean line. * *p* < 0.05.

Furthermore, the magnitude of interlimb transfer was similar to the amount of motor skill acquisition at each level (Fig. 5) indicated by a non-significant main effect of hand (motor skill acquisition of the practicing dominant hand, interlimb transfer to the non-dominant hand) (F_1,24_ = 0.361, *p* = 0.554, η_p_^2^ = 0.015) and a non-significant hand × sequence complexity interaction (F_3,22_ = 0.720, *p* = 0.551, η_p_^2^ = 0.089). Post-hoc analyses revealed that participants’ RT improved the most at the most complex finger tapping sequence task for both motor skill acquisition and interlimb transfer, regardless of handedness.

### Changes in behavioral data during the motor sequence practice (results of practice blocks)

Neither block main effect (F_4,20_ = 2.543, *p* = 0.072, η_p_^2^ = 0.337), nor handedness × block interaction effect (F_4,20_ = 2.026, *p* = 0.129, η_p_^2^ = 0.288) was found in NASA-TLX suggesting that participants subjectively experienced a similar mental workload throughout the 5 blocks of motor sequence practice. In line with this, participants performed the 5 blocks of motor sequence practice with similar accuracy, indicated by non-significant block main effect (F_4,20_ = 2.530, *p* = 0.073, η_p_^2^ = 0.336) and non-significant handedness × block interaction effect (F_4,20_ = 1.737, *p* = 0.181, η_p_^2^ = 0.258).

Significant block main effect was found in RT (F_4,20_ = 12.915, *p* = 0.073, η_p_^2^ = 0.721) with the post-hoc test revealing improved RT between the first (*p* < 0.001, d = 1.033) and last (*p* = 0.005, d = 1.962) 2 blocks, regardless of handedness. Nevertheless, these improvements in RT were not associated with lower mental workload (all *p* > 0.05).

## Discussion

This study is the first to examine a potential interaction between handedness and finger key press sequence complexity. Both right- and left-handed participants performed the motor sequence task with faster RT when using their right hand which may indicate a left-hemisphere specialization for motor sequencing tasks. The main findings of the present study are that the motor sequence practice produced both motor skill acquisition and interlimb transfer indicated by a decreased reaction time of finger key presses, however, these changes were independent of handedness. However, participants performed the motor sequence tasks less accurately at levels 3 and 4 after versus before practice. Higher sequence complexity resulted in longer RT, however, both right- and left-handed participants’ RT increased with the same magnitude with the increase in sequence complexity. Similar behavioral pattern was observed in MT as in RT most probably because pressing a key on a keyboard is considered a rather simple motor task with most resources linked to the planning of the movement but not for the movement execution itself, therefore, only the planning part of pressing the appropriate keys increased with increasing sequence complexity. Finger sequence complexity also affected the perceived mental workload as measured by the NASA-TLX. Overall, in line with our hypothesis, participants’ motor sequence performance was better with their right hand, irrespective of handedness, however, handedness did not affect motor skill acquisition by the dominant hand or interlimb transfer to the non-dominant hand regardless of task complexity level.

### Potential left-hemisphere specialization for motor sequencing tasks

Early clinical neuroimaging studies suggested that after left but not right hemispheric damage, motor skills were substantially impaired^25,46,47^. These data implied that the left-hemisphere might be a dominant controller of motor sequencing skills. However, more recent literature has brought into question the notion of hemispheric motor lateralization considering that imaging and brain stimulation studies falsified the hypothesis that the left hemisphere is dominant for controlling simple or complex movements^48–50^. In fact, a symmetrical bilateral activation pattern could be observed in the sensorimotor^51,52^ and rostral prefrontal cortex^52–54^ areas during unilateral handgrip tasks. There is also evidence that task complexity level increases the activity of the ipsilateral M1^34,55^, while larger handgrip force results in increased ipsilateral sensorimotor activation and greater functional connectivity between hemispheres within the sensorimotor network^50^. Findings of the present study show better motor sequence performance of the right hand, indexed by faster RT, in both right (right hand: 0.88 ± 0.32 s, left hand: 0.94 ± 0.34 s; d = 0.182) and left-handed participants (right hand: 1.02 ± 0.39 s, left hand: 1.07 ± 0.4 s; d = 0.127). Although findings of previous clinical studies^31–33^ also indicated a potential association between the left hemisphere and higher-order aspects of motor control, we did not find handedness × sequence complexity interactions. In addition, although the number of correct motor sequences decreased with the increase in sequence complexity, this phenomenon was independent of handedness. Overall, although the present study lacks mechanistic neurophysiological data to support the findings of motor lateralization to the left hemisphere, hemispheric lateralization for motor sequencing tasks seems to be apparent based on our behavioral data, however, the nature of the differences between the two hands was unrelated to the level of sequence complexity.

### Performance changes in response to practice are independent of handedness

In the present study, we aimed to examine whether a 5 blocks × 60 trials motor sequence practice with the participants’ dominant hand would result in motor skill acquisition and interlimb transfer. Moreover, we also aimed to determine whether these changes were influenced by handedness or sequence complexity. The literature is controversial regarding the effects of handedness on motor skill acquisition. For example, in a previous study, right-handed people demonstrated significantly greater skill improvements for their dominant right versus non-dominant left hand, however, left-handers demonstrated comparable learning effects in each hand^20^. These results suggest a potential influence of handedness on the hemispheric specialization of motor skill learning. In contrast, results of a previous study^21^ showed a significant improvement of motor skill performance in right-handed individuals only when they practiced the grooved pegboard task with their non-dominant left hand. Nevertheless, we also found similar magnitude of learning in each hand of left-handed people.

In the present study, we hypothesized that a dominant-hand practice would result in higher magnitude of motor skill acquisition in right-handed participants. We found motor skill acquisition indicated by a decreased RT at each complexity levels and a decreased MT at levels 1, 2, and 4, however, these changes were independent of handedness suggesting that the magnitude of motor skill acquisition in response to dominant-hand motor skill practice were similar in right and left-handed people. Based on a previous study^35^, we expected to find differences between left and right-handed participants’ performance at different sequence complexities in response to motor sequence practice, however, we found no handedness × sequence complexity interaction effect. The lack of interactions between sequence complexity and handedness might be explained by three reasons. First, the level of difficulty between the 4 complexity levels might not have been significantly different from each other so that the more difficult conditions were challenging enough to detect differences between left and right-handed individuals, if any, at higher complexity levels. Another possible reason for the lack of a handedness x complexity interaction in the present study may be related to the complexity of the motor aspect of sequence learning tasks. Specifically, pressing a key on a keyboard is considered a rather simple motor task with most resources linked to the planning of the movement, i.e., pressing the correct button, but not for the movement execution itself. Thus, although we applied 4 levels of sequence complexity in the present study, the increase in sequence complexity might have not required an increase in motor control, considering that the key-pressing movement itself remained monotonic, unchanged during the task. Lastly, we recruited healthy young participants in our study and did not apply the experimental conditions on elderly adults or patients undergoing rehabilitation after a left or right-hemisphere stroke. Future work is needed to confirm the hypothesis of whether interactions could be found between handedness and motor sequencing tasks with different complexity levels in aged adults or neurological patients after unilateral damage by a stroke after practicing a motor sequence skill with the dominant or non-dominant hand.

Even though changes in accuracy (% of correct sequences) were also independent of handedness, a time × sequence complexity interaction and post-hoc tests showing significant decrease in accuracy at levels 3 and 4 indicated that each participant made more errors at higher task difficulties at the end of the ~ 120 min experiment most probably due to mental fatigue. Nevertheless, accuracy and participants’ subjective experience of mental workload were significantly associated only at level 3 (r = − 0.509, *p* = 0.009). We considered whether participants performed the motor sequence tasks with more errors at higher task difficulties because they wanted to perform the task with better reaction- and movement times. Therefore, statistical analyses were also performed with only the correct answer’s RT and MT. In each case, results were almost the same as the results of the analyses of both correct and incorrect trials suggesting that the decrease in accuracy at levels 3 and 4 were most probably due to participants’ mental fatigue. However, participants’ decreased RT was not associated with participants’ subjective mental workload neither for the practicing dominant nor for the non-practicing non-dominant hand. It is, therefore, unclear how participants’ mental fatigue affected motor sequencing accuracy but not RT.

Because previous studies failed to detect the role of right versus left-hand dominance in cross-education, we were also unable to propose a directional hypothesis regarding the effect of handedness on interlimb transfer to the non-dominant hand after dominant hand motor sequence practice. In the present study, the practice induced interlimb transfer indicated by a decreased reaction time at levels 1, 2, and 4 and a decreased movement time at each complexity level, however, these changes were also independent of handedness suggesting that the magnitude of interlimb transfer were similar in right and left-handed participants. We hypothesized that greater cognitive processing might be needed for more complex tasks, thus, the level of complexity could act as stimulus for skills acquisition and for its transfer as well. Indeed, we found that the magnitude of interlimb transfer was similar to the amount of motor skill acquisition at each level (Fig. 4B), regardless of handedness. Specifically, participants’ RT improved the most at the most complex finger tapping sequence task for both motor skill acquisition (pre: 1.44 ± 0.3 s; post: 1.24 ± 0.3 s, d = 0.667) and interlimb transfer (pre: 1.47 ± 0.3 s; post: 1.31 ± 0.3 s, d = 0.533). A previous study showed better interlimb transfer of sensorimotor adaptation in less strongly left-handed individuals, regardless of the practicing hand, and better interlimb transfer of sequence learning in less strongly right-handed individuals after dominant hand practice^27^. Furthermore, another study^28^ concluded that the magnitude of interlimb transfer could be predicted based on the participant's task performance and laterality quotient suggesting a strong association between the degree of handedness and the magnitude of interlimb transfer. Nevertheless, in the present study, we recruited only strongly left (LI: –89.1 ± 11.4) and strongly right-handed (LI: 88.4 ± 11.5) participants to control this possible association.

We not only examined the performance of participants before and after practice but also considered the rate of improvement in performance during the practice. Our results indicate that participants performed the 5 blocks of motor sequence practice with similar accuracy. This was supported by the NASA-TLX data revealing that participants subjectively perceived a similar mental workload throughout the 5 blocks of motor sequence practice. Although improved RT and MT were found between the first (*p* < 0.001, d = 1.033) and last (*p* = 0.005, d = 1.962) 2 blocks, regardless of handedness, this might have been due to the motivation of the participant. Nevertheless, the improvements in RT were not associated with lower mental workload. This indicates that participants’ subjective experience on task complexity during the practice was aligned with the experimental design considering that the level of sequence complexity was the same in each block, only the appearance of the same sequences was randomized.

### Associations between motor sequence performance and participants’ experience on mental workload

We supplemented our behavioral data with the National Aeronautics and Space Administration Task Load Index (NASA-TLX) to determine if participants subjective rating of task complexity aligned with the experimentally designed sequence complexity. NASA-TLX is used in a wide spectrum e.g. to assess surgeons' intraoperative cognitive workload^56^, to monitor the mental workload in geriatric inpatients during an exergame-based cognitive-motor training program^57^ or to identify the association between several contextual match factors, technical performance, and external movement demands on the subjective task load of elite rugby league players^58^.

In the present study, participants’ decreased RT was not associated with participants’ subjective mental workload neither for the practicing dominant nor for the non-practicing non-dominant hand. However, mental workload increased with the increase in sequence complexity indicating that participants’ subjective judgment on their mental workload was associated with their performance or with task complexity. This is in contrast with the findings of a previous study^59^ reporting no effects of task complexity on perceived mental workload. A possible reason for these contradictory results can be related to the different tasks applied in the two studies considering that the previous used not a finger sequence task but a visuomotor task. It is also important to mention that NASA-TLX was performed at the end of each block of both the motor sequence tests and practice, therefore, it significantly increased the time needed for the experiment. Overall, NASA-TLX appeared to be a useful tool for the subjective assessment of participants’ mental workload, its most appropriate timing of application must be carefully considered especially in longer experiments or in those with neuroimaging recordings.

### Clinical perspectives

Clarifying how the interaction between handedness and the complexity of the motor sequence task, if any, could affect motor skill acquisition and interlimb transfer could improve the efficacy of rehabilitation of patients with unilateral neurological or orthopedic impairments. In the present study we found increasing RT with increasing sequence complexity level, this association was unrelated to handedness. Handedness did not affect motor skill acquisition by the dominant hand or interlimb transfer to the non-dominant hand, however, this might be due to the health condition of our participants. It is possible that even the most complex sequence level was not challenging enough to reveal differences between healthy left and right-handed participants, however, applying our experimental conditions on elderly adults or patients undergoing rehabilitation after a left or right-hemisphere stroke might have provided different results. During the period after injury when one limb is unable to function temporarily or chronically due to a brain damage caused by a stroke, rehabilitative interventions showing large degree of interlimb transfer can be usefully applied^60^. Motor skill acquisition and interlimb transfer known to continue operating despite the prolonged absence of proprioceptive and visual feedback even in upper-limb amputees^61^, however, it is still unknown whether right and left-handed patients would need to do the same rehabilitation differently for a higher efficacy after left or right-hemisphere damage for a higher magnitude of interlimb transfer. While some^62,63^ but not all^64^, studies report higher frequency of strokes in the left hemisphere, our results might have implications to predict the recovery of manipulative skills based on stroke location and handedness. Notably, it is important to distinguish between manipulative, visuomotor, and motor sequence learning skills considering that manipulative skills require more complex motor components, such as reaching, grasping, and manipulating tools. Altogether, future work is needed to confirm the hypothesis of whether interactions could be found between handedness and motor sequencing tasks with different complexity levels in aged adults or neurological patients after unilateral damage by a stroke after practicing a motor skill with the dominant or non-dominant hand.

### Limitations and future perspectives

The limitation of the present study is the lack of neurophysiological data. Although our RT results may indicate a left-hemisphere specialization for motor sequencing tasks, thus supporting the findings of previous clinical studies reporting a significant decline in motor skills after left but not right hemispheric damage^25^, future studies should determine the potential underlying mechanisms of the interactions between handedness and sequence complexity i.e., which areas within each hemisphere are responsible for such interaction during a motor sequencing task using EEG or fMRI. Moreover, it would also be important to examine the effects of age on this interaction. Another limitation of the present study is related to the lack of non-dominant hand practice. Because our crossover study design did not involve the practice of the non-dominant hand, we cannot draw clear conclusions whether handedness would affect motor skill acquisition and interlimb transfer. Nevertheless, when performing such a study, one should carefully consider the time needed to ‘wash out’ the effects of the first practice, regardless of whether it was first performed with the dominant or non-dominant hand. Furthermore, because manipulative skill tasks often require more complex motor components (such as reaching, grasping, manipulating tools), perhaps a keypress-based motor sequence learning task should not be considered a manipulative task. Moreover, although our pre-statistical power analysis indicated that a total sample size of 22 would be appropriate to detect differences between the groups, the sample size was justified based on an a priori power analysis using the effect size from our previous study^21^ that recruited 24 participants. However, because small samples can often lead to overestimated effect sizes^65,66^, future studies should justify the sample size based on power analysis drawing on multiple prior studies or a meta-analysis. Lastly, considering that motor memory consolidation is sleep-dependent, future studies should perform a retention test 24 h after the practice to clearly identify weather handedness interacts with motor sequencing tasks with different complexity levels after motor sequence practice.

## Conclusions

In conclusion, both right- and left-handed participants performed the motor sequence task with faster RT when using their right hand. In line with this, right-handed participants performed the motor sequence task faster with their dominant versus non-dominant hand. Motor sequence practice produced both motor skill acquisition and interlimb transfer indicated by a decreased reaction time of finger key presses at different sequence complexity levels, however, these changes were independent of handedness. However, participants performed the motor sequence tasks with less accuracy at levels 3 and 4 after the practice as compared to the pre-test. Similar behavioral pattern was observed in MT as in RT. Overall, in line with our hypothesis, our results may indicate left-hemisphere specialization for motor sequencing tasks, however, neuroimaging studies are needed to support these findings. On the other hand, handedness did not affect motor skill acquisition by the dominant hand or interlimb transfer to the non-dominant hand regardless of task complexity level.

## Supplementary Information

Supplementary Information.

## Data Availability

The datasets used and/or analyzed during the current study are presented within the manuscript and/or additional supporting files, and also available from the corresponding author on reasonable request.

## Abbreviations

ANOVA: Analysis of variance
D: Dominant hand
L: Sequence complexity level
LH: Left-handed participants
LI: Laterality index
MT: Movement time
NASA-TLX: National Aeronautics and Space Administration Task Load Index
ND: Non-dominant hand
RH: Right-handed participants
RT: Reaction time
