# Supplementary material for: Handedness did not affect motor skill acquisition by the dominant hand or interlimb transfer to the non-dominant hand regardless of task complexity level
Source: Sci Rep. 2022 Oct 28;12:18181. doi: 10.1038/s41598-022-21962-2 (PMC9616877; doi:10.1038/s41598-022-21962-2)
Supplement: Supplementary file 1 — Supplementary Information. [file 41598_2022_21962_MOESM1_ESM.docx]

*Supplementary material*

**Title:** Handedness did not affect motor skill acquisition by the dominant hand or interlimb transfer to the non-dominant hand regardless of task complexity level

**Journal name:** nature Scientific Reports

**Authors:** János Négyesi^1, *^, Péter Négyesi^2^, Tibor Hortobágyi^3, 4, 5, 6^, Sai Sun^7, 8^, Joji Kusuyama^1, 7^, Rita M. Kiss^9^, Ryoichi Nagatomi^1, 10^

**Affiliations:** ^1^Division of Biomedical Engineering for Health and Welfare, Tohoku University Graduate School of Biomedical Engineering, Sendai, Japan; ^2^Doctoral School of Education, Eszterházy Károly Catholic University, Eger, Hungary; ^3^Department of Kinesiology, Hungarian University of Sports Science, Budapest, Hungary; ^4^Institute of Sport Sciences and Physical Education, University of Pécs, Pécs, Hungary; ^5^Somogy County Kaposi Mór Teaching Hospital, Kaposvár, Hungary; ^6^Center for Human Movement Sciences, University of Groningen, University Medical Center Groningen, Groningen, The Netherlands; ^7^Frontier Research Institute for Interdisciplinary Sciences, Tohoku University, Sendai, Japan; ^8^Research Institute of Electrical Communication, Tohoku University, Sendai, Japan; ^9^Faculty of Mechanical Engineering, Department of Mechatronics, Optics and Engineering Informatics, Budapest University of Technology and Economics, Hungary; ^10^Department of Medicine and Science in Sports and Exercise, Tohoku University Graduate School of Medicine, Sendai, Japan

**Corresponding author:**

János Négyesi

e-mail: negyesi@tohoku.ac.jp


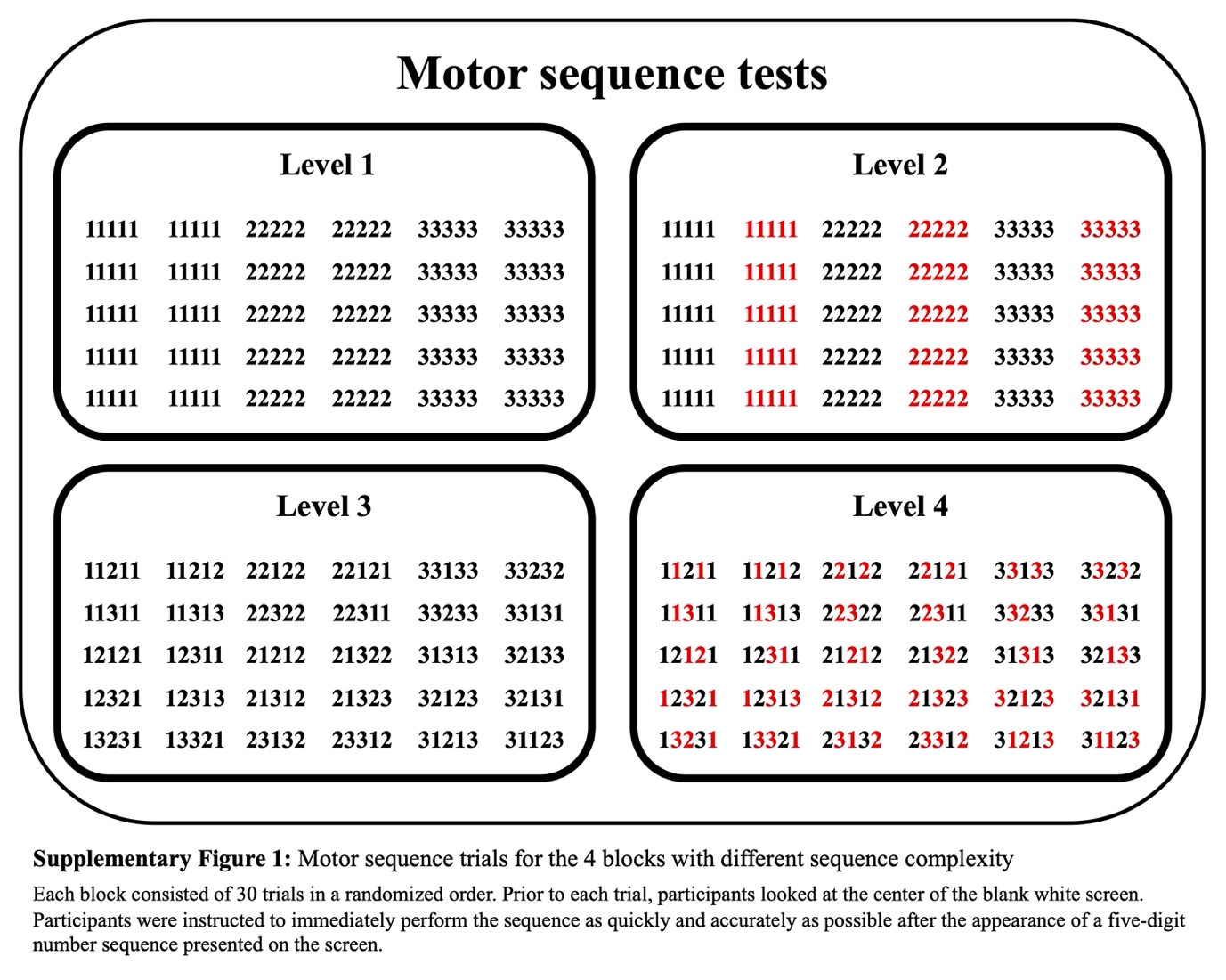


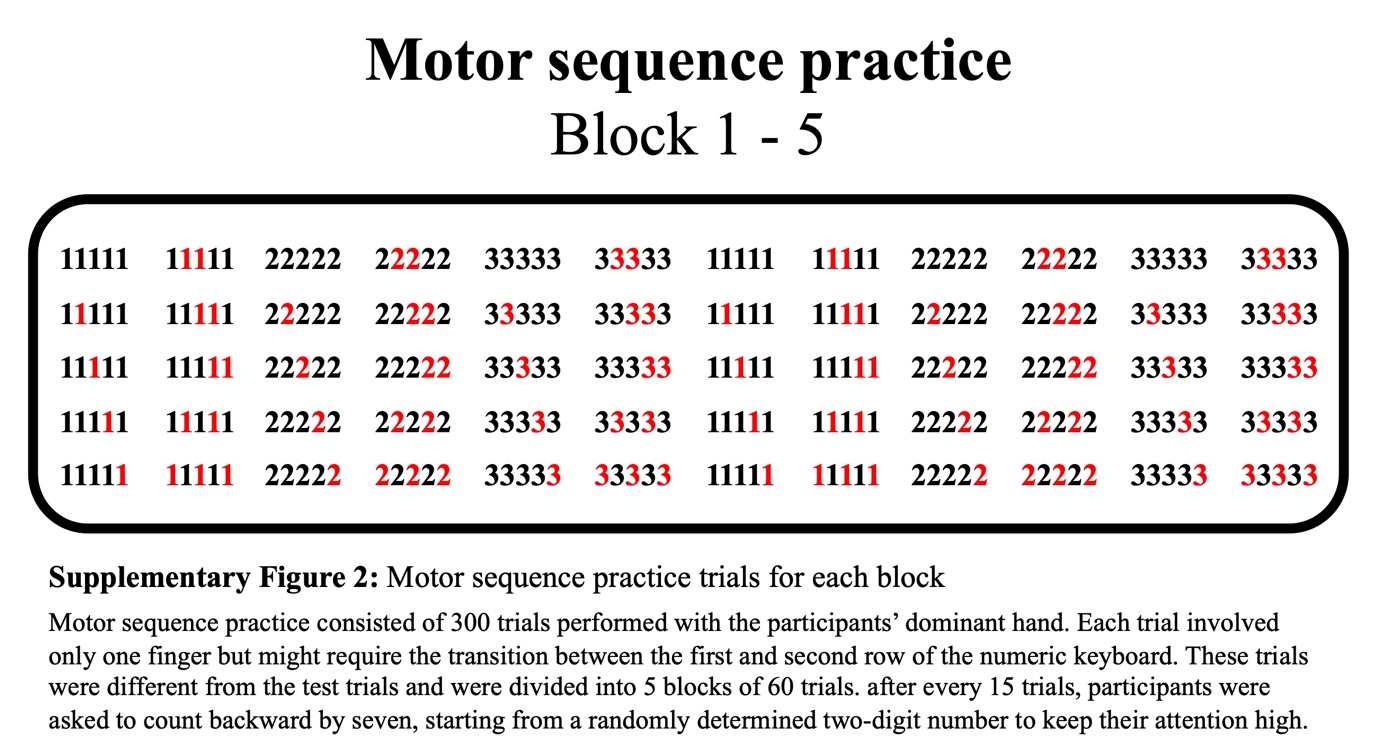


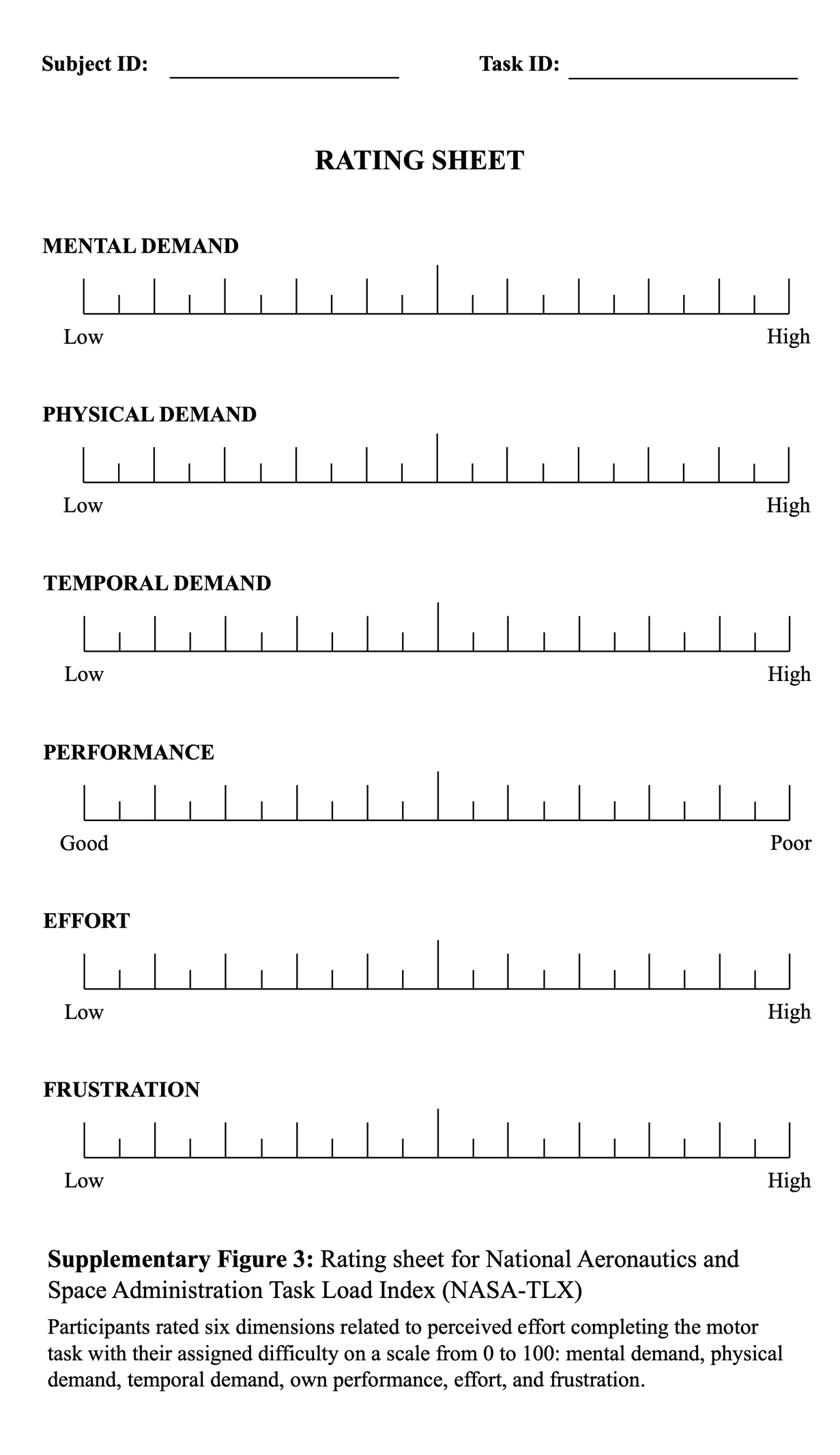


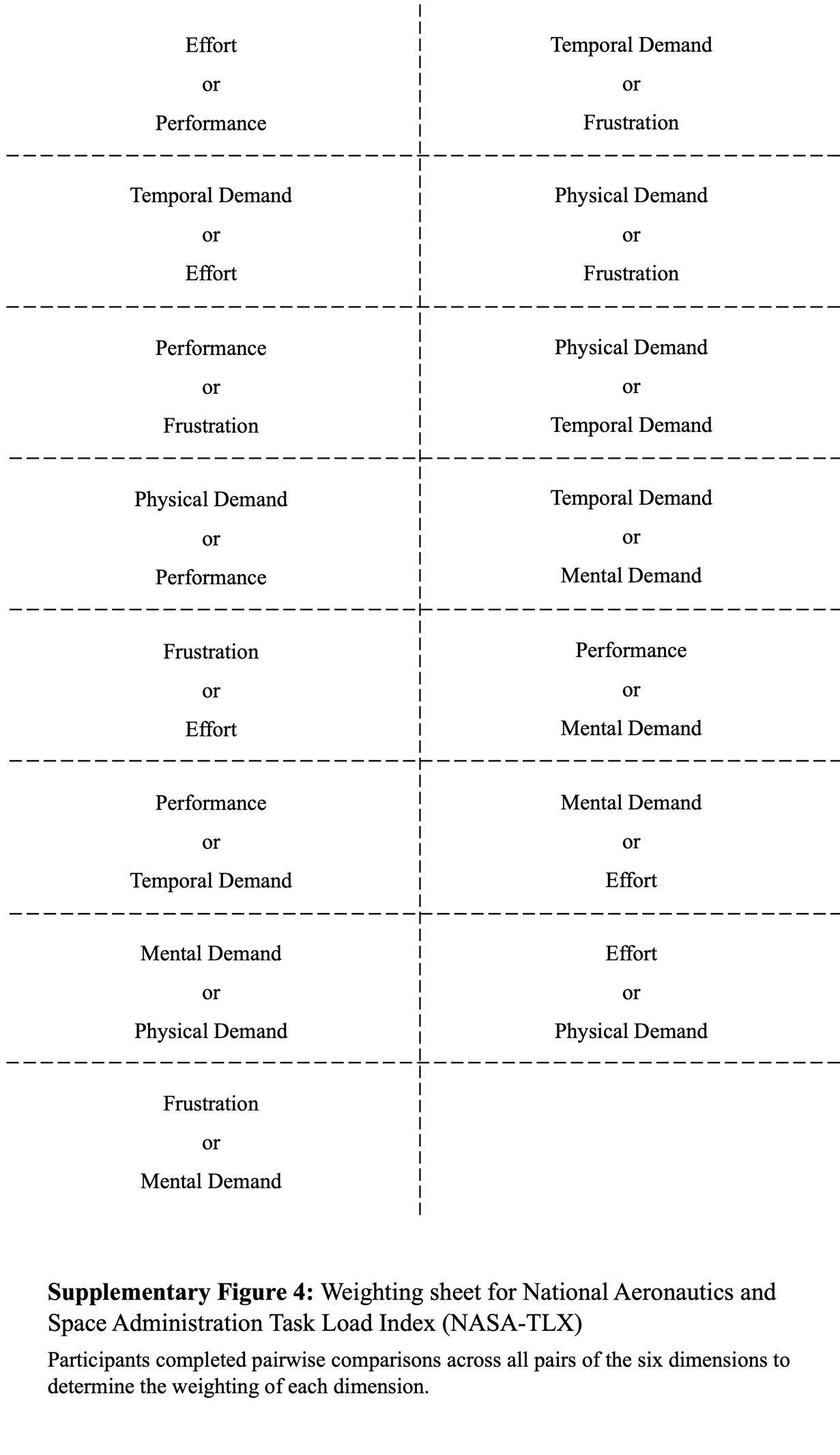


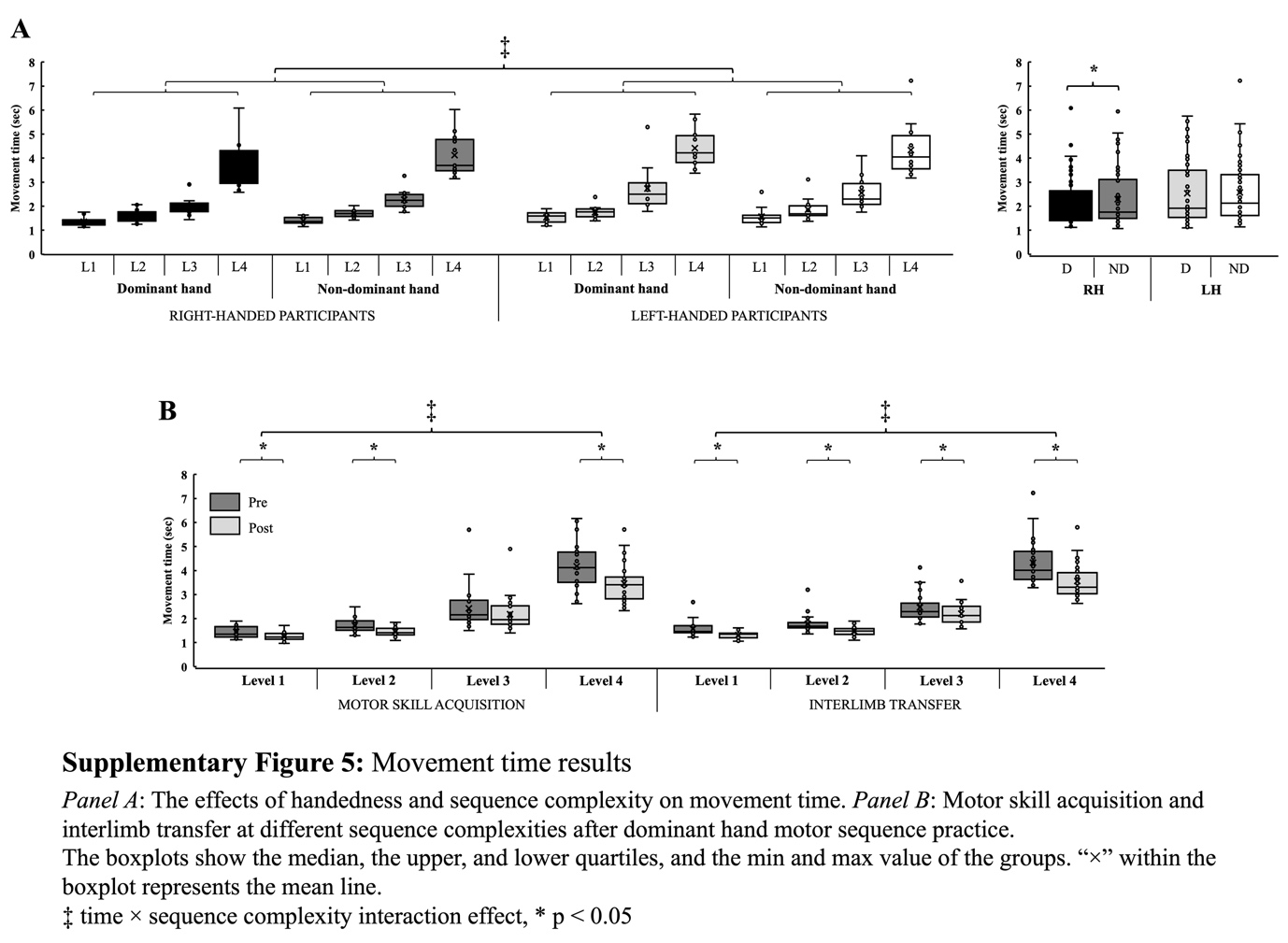


| **Supplementary Table 1:** Results of statistical analyses regarding movement time of the finger sequence task | | | | | | |
| --- | --- | --- | --- | --- | --- | --- |
| Differences between left and right-handed participants during the motor sequence test | | | | | | |
| *main effect* |  | F |  | p |  | η_p_^2^ |
| task complexity |  | 250.6 |  | < 0.001 |  | 0.97 |
| hand |  | 4.9 |  | 0.037 |  | 0.18 |
| *interaction effect* |  |  |  |  |  |  |
| handedness x hand |  | 7.9 |  | 0.010 |  | 0.26 |
|  |  |  |  |  |  |  |
| Motor skill acquisition and interlimb transfer at different task complexity after dominant hand motor sequence practice | | | | | | |
| Motor skill acquisition |  | F |  | p |  | η_p_^2^ |
| *main effect* |  |  |  |  |  |  |
| time |  | 86.8 |  | < 0.001 |  | 0.79 |
| task complexity |  | 303.2 |  | < 0.001 |  | 0.98 |
| *interaction effect* |  |  |  |  |  |  |
| time x task complexity |  | 4.2 |  | 0.018 |  | 0.38 |
|  |  |  |  |  |  |  |
| Interlimb transfer |  |  |  |  |  |  |
| *main effect* |  |  |  |  |  |  |
| time |  | 79.6 |  | < 0.001 |  | 0.78 |
| task complexity |  | 423.2 |  | < 0.001 |  | 0.98 |
| *interaction effect* |  |  |  |  |  |  |
| time x task complexity |  | 5.8 |  | 0.005 |  | 0.45 |
|  |  |  |  |  |  |  |
| Changes in behavioral data during the motor sequence practice | | | | | | |
| main effect |  | F |  | p |  | η_p_^2^ |
| block |  | 19.5 |  | < 0.001 |  | 0.80 |

**Supplementary Table 2:** Supplementary dataset for absolute values of accuracy

**Supplementary Table 3:** Supplementary dataset for reaction time (RT)

**Supplementary Table 4:** Supplementary dataset for reaction time (RT) of correct trials

**Supplementary Table 5:** Supplementary dataset for movement time (MT)

**Supplementary Table 6:** Supplementary dataset for movement time (MT) of correct trials

**Supplementary Table 7:** Supplementary dataset for NASA-TLX
